# Implementation and impact of the global access principles at the University of British Columbia: current successes and future challenges

**DOI:** 10.3389/fphar.2023.1271618

**Published:** 2023-12-15

**Authors:** Nancy Gallini, Robert E. W. Hancock, Richard Lester, Brett Sharp, Kishor M. Wasan

**Affiliations:** ^1^ Vancouver School of Economics, The University of British Columbia, Vancouver, BC, Canada; ^2^ The Neglected Global Diseases Initiative, The University of British Columbia, Vancouver, BC, Canada; ^3^ Department of Microbiology and Immunology, Faculty of Science, The University of British Columbia, Vancouver, BC, Canada; ^4^ Department of Medicine, Division of Infectious Diseases, The University of British Columbia, Vancouver, BC, Canada; ^5^ University-Industry Liaison Office, The University of British Columbia, Vancouver, BC, Canada; ^6^ Department of Urologic Sciences, Faculty of Medicine, The University of British Columbia, Vancouver, BC, Canada

**Keywords:** global access principles, neglected diseases, essential medicines, infectious diseases, telemedicine

## Abstract

In 2007, the University of British Columbia (UBC) was the first university in Canada to establish and adopt global access (GA) principles. Toward implementing these principles, UBC then identified a set of strategies for providing affordable access to new UBC-developed technologies throughout low- and middle-income countries and among vulnerable populations. In this perspective, we provide an update of UBC’s progress over the past 15 years made on several technologies that fall under the GA principles. The technologies reported on are wide-ranging, including an oral medication for the treatment of leishmaniasis; peptides for potential use against malaria, and various bacterial, viral and fungal infections; a portable vaccine cooler; a diagnostic technology to detect severe sepsis; and an SMS Messaging System to monitor and support patients with HIV, TB and COVID-19. We identify challenges faced by the researchers in implementing the GA principles for these technologies and potential solutions for overcoming them through creative licensing and partnerships with public and private sectors, governments, local companies, and communities. As the UBC example illustrates, universities across the globe have an opportunity to make a significant social impact on improving global health of vulnerable populations and on supporting local infrastructures for sustaining these improvements.

## 1 Introduction

In 2009, the *Journal of Pharmaceutical Sciences* published a commentary on the Global Access (GA) Initiative at the University of British Columbia (UBC) that was created in 2007. As noted in the article, UBC was the first university in Canada, in collaboration with the Universities Allied for Essential Medicines (UAEM; UBC Chapter), to develop and adopt a set of global access principles and strategies (hereafter, the GA principles) for research, licensing and patenting of new innovations, aimed at providing affordable access to UBC-developed technologies, at or below development cost, throughout low- and middle-income countries (LMIC) and among vulnerable populations.^1^ Shortly after, UBC endorsed the Statement of Principles and Strategies for the Equitable Dissemination of Medical Technologies, which supplemented the UBC principles.^2^ Based on the actions taken by UBC and its researchers at the initial phase of implementation, UBC was heralded as a global access leader both in its research on global health innovations and its commitment to provide affordable access to those innovations (Guebert and Bubela, 2014).

In the first section of this perspective, we present an update of UBC’s progress made in implementing the GA principles and identify the barriers faced and potential solutions for overcoming those barriers. In Section 2 we re-affirm UBC’s ongoing commitment, and the significant progress made toward fulfilling its GA mandate. In Section 3 we provide progress updates over the past 15 years for technologies reviewed in the 2009 commentary and present new technologies that have been developed under those principles in recent years. In Section 4 we address the challenges faced by the researchers in the previous section, suggesting that these, among others, may explain the modest pace at which the GA principles have been implemented globally, as reported in Nguyen, Shahzad, Veras (2018). Section 5 concludes with a call for a revival of universities’ commitments to the GA principles.

## 2 UBC’s commitment to the GA principles

The principles, presented in the 2009 commentary, can be summarized in two action items:

(1) Engage in research and development on technologies that will benefit LMIC; and
(2) Provide global access to research tools^3^ and health-related products developed at UBC.

For UBC to maximize the global impact from its technologies, subject to economic constraints, several strategies were identified in the commentary. In particular, to support research and develop new technologies that are environmentally sustainable, respect biodiversity and benefit LMIC, UBC partnered with companies, foundations and non-profits. To support global access to the technologies from this research, UBC has employed a variety of licensing practices in low and middle-income countries, including non-exclusive licensing of research tools, field-of use limitations on exclusive licenses of medical products, cost-based pricing and restrictions on patenting to, ensure locally-affordable pricing for technologies, and support for a sustainable local infrastructure. The high cost of research and development and commercialization requires that economic and financial considerations be part of successful implementation of the GA principles. Nevertheless, maximization of social impact was and continues to be at the core of UBC’s implementation strategy.

The GA principles, identified 15 years ago, continue to guide UBC in its research and access to medical technologies developed at UBC. In fact, UBC has strengthened its commitment to the these principles by the following additional actions:

i. UBC, through the University-Industry Liaison Office (UILO), takes a broad perspective on research and access to its medical innovations that focus on societal benefits and vulnerable populations.^4^ As UBC policy only requires UBC researchers to engage with the UILO if they intend to patent, license or sell university research products, the UILO does not see the majority research activities conducted at UBC, many of which have potential societal impacts that are far-reaching but are not appropriate for commercial mobilization.^5^ However, for those innovations that are disclosed for commercial or non-commercial licensing, the UILO now includes the evaluation of potential social impact in its technology assessment process. So, for example, if an innovation has low financial value but potentially high social impact (health, environment, security), UILO will work with the researchers to try and find a path to mobilize the technology.^6^
ii. Currently, there are at least a dozen projects at UBC and our Affiliated Hospitals that are being mobilized according to UBC’s GA principles, which is significantly higher than 15 years ago. These include a wide range of drugs and medical devices. A subset of those research projects is discussed in Section 3. Another cohort of research projects does not explicitly contain GA relevant language but have been structured in a manner that maximizes public good.^7^
iii. The Neglected Global Disease Initiative (NGDI), which provided the foundational research leadership of UBC’s GA principles, has been very active in research collaborations and outreach activities. It has also prioritized educating the next generation of global health researchers through student involvement. Educating and engaging students has become increasingly central to UBC’s priority of Global Health through the Faculty of Medicine, the School of Population and Public Health, NGDI and other academic units within the University.
iv. In the past 2 years, UBC’s UILO has expanded implementation of its GA principles into emerging diseases by additionally signing onto the AUTM COVID-19 Licensing Guidelines.^8^ Subsequently, in response to the Covid pandemic, UBC entered into license agreements, consistent with the AUTM guidelines, with new researcher-founded spin-off companies such as Eyam Vaccines Immunotherapeutics Ltd. and Sepset Biosciences Inc., described in the next section.

## 3 Research projects under the global access principles

In this section, we report on the progress over the past 15 years of several research projects that fall under the GA principles, including two highlighted in the 2009 commentary: Dr. Kishor Wasan’s laboratory working on a novel oral Amphotericin B formulation for the treatment of leishmaniasis and systemic fungal infections and Dr. Bob Hancock’s research on new (immunomodulatory) treatments of antibiotic resistant infections impacting LMIC world-wide.

The oral amphotericin B formulation invented by Drs. Ellen and Kishor Wasan is one of the signature technologies under the UBC GA Principles. The Wasan lab committed to providing the technology at or below manufacturing costs for indications in LMIC and diseases including leishmaniasis. This oral amphotericin B formulation, which has received funding from both the Canadian Institutes of Health and the Consortium for Parasitic Drug Development (a Gates Foundation Grantee), is cost-effective, safe, tropically stable, accessible and with positive human phase 1a/1b safety data. Formulation, pre-clinical pharmacology and GLP toxicology work, which supported orphan drug designation have been completed. The formulation has received positive support from MSF-Canada, MSF-Korea, the MSF NTD Working Group, the WHO Working Group. Recently, the oral amphotericin B formulation has recently been listed on the WHO priority list in the treatment of children with visceral leishmaniasis as it would address the limitations of current treatments. (https://www.who.int/publications/i/item/9789240085176). Moreover, it is currently being reviewed by several global health groups for potential phases 2/3 human efficacy studies in patients with visceral leishmaniasis in several regions around the world.

Peptides discovered by the Hancock lab were shown to have potent immunomodulatory activity and were initially licensed to Inimex Pharmaceuticals, a UBC spinoff that developed SGX942. This peptide was eventually sublicensed to US company Soligenix, which showed the molecule’s efficacy in a phase 2 trial against oral mucositis in cancer patients. While it has not been pursued for any developing country disease, efficacy in animal models of malaria, pre-term birth, and various bacterial, viral and fungal infections have been demonstrated for 2nd generation peptides like IDR1018^9^. Related peptides also worked well as components of adjuvant formulations for vaccination in single dose without maternal interference in neonatal pigs and mice. These peptides were licensed to a private UBC spinoff company, ABT Innovations Inc., which is developing peptides for these uses as well as anti-biofilm infection agents. ABT Innovations and Sepset Biosciences Inc., mentioned above, are now majority-owned by the public company Asep Medical Inc. that is committed to global access.

Over the past 15 years, several new projects have been particularly relevant to the GA principles. Two spin-off companies Birch Biomedical, a clinical stage biomedical company focused on the clinical development and commercialization of fibroproliferative therapy products, and CoMotion Drug Delivery Systems, an early-stage company developing advanced hemostatic therapeutics, have entered into license agreements with UBC that require them to make relevant products available in LMIC that is consistent with UBC’s GA principles. Furthermore, in partnership with the Clinton Health Access Initiative, UBC has licensed the Portable Vaccine Cooler technology to Novare Corporation for deployment in LMIC.

In response to the COVID-19 pandemic, the license with Sepset noted above enabled the sharing with poorer nations of technology relevant to the diagnosis of severe sepsis, which is the cause of most deaths due to life-threatening Covid (Beghela, et al., 2022; Baghela, et al., 2023). Moreover, the technology allows for the separation of sepsis patients into mechanistic subtypes termed endotypes (Baghela, et al., 2023). This means that sepsis, now known to annually cause nearly 20% of all deaths on the planet (Rudd, et al., 2002), can be treated in an endotype-specific manner. In particular, the patient is assigned, using a simple diagnostic, to a particular endotype, dictating the types of medicines that will work against their type of sepsis.

Another cohort of agreements or relationships do not explicitly contain GA relevant language but were structured in a manner that maximizes public good. An excellent example is WelTel Incorporated, a Canadian registered software company cofounded by Dr. Richard Lester. WelTel is an independent company whose technology was developed in the course of UBC research. Given the societal-good mission of WelTel, and in order to minimize barriers to the success of the enterprise, UBC assigned its interest in the technology to the company at no cost. WelTel developed and deployed an SMS Messaging System to monitor and support patients with HIV and other conditions in North American and Africa^10^ and has been shown to be cost-effective. Patel, et. al. (2017) In addition, WelTel’s technology was adopted nationally by the Rwandan government for COVID-19 monitoring and support, then for Ebola border surveillance during the recent outbreak in Uganda. WelTel is working with UBC on collaborations around data science and artificial intelligence (AI) using natural language processing (NLP) to learn from patients’ experiences and improve patient-centered care^11^ and with the Rwandan government and its partners (USAID, Rwanda Biomedical Center, and African Center for Excellence in Data Science) to adopt WelTel’s products at the national scale for public health (Babili, Nsanzimana, Rwagasore, Lester, 2023).

Using WelTel’s products, the Rwanda government identified a gap in how outpatients are followed in general as they strive along with the United Nations Sustainable Development Goals (SDGs) to reimagine primary healthcare to be more efficient, available, and responsive to its citizens. For long-term evaluation, the WelTel programs continue their contact with patients after they leave healthcare facilities and return to the community, so many of their problems can be triaged or solved remotely. This saves time and money for patients and may help declog some health services. Discussions are currently taking place with the Rwandan government over implementing the WelTel technology as core digital health tool in the national health programs for everything from chronic diseases, mental health (post-genocide), and maternal child health. As Rwanda is a regional leader in digitalization^12^, adopting the technology nationally will provide opportunities for surrounding countries.

WelTel is at the forefront of digital health technologies that are becoming an increasingly important pipeline of innovations as the start-up overhead can be lower than wet lab developments. Moreover, the plethora of AI-enabled development tools makes putting ideas into practice more accessible for students and domain experts with a socio-entrepreneurial spirit.

## 4 Challenges

Implementing the GA principles requires a delicate balance between attracting economic support for research and development of new innovations and ensuring fair and affordable access to those technologies in LMIC (Bottazzi and Hotez, 2019). The projects outlined in Section 3, while having made significant progress in achieving this balance, had to overcome several economic, bureaucratic, and political hurdles that may have delayed or compromised implementation of the GA principles altogether.

Researchers at UBC spoke of two types of overarching challenges. First, while grant funding is often available in the discovery stage of research, acquiring public and private funding to sustain later stages of research and development (e.g., clinical trials, FDA approval, product launch and scale-up) is considerably more difficult. Nevertheless, new funding models involving product development partnerships with international collaboration platforms, non-profit organizations, private foundations and donors, and federal and local government are increasingly available (Bishai, et al., 2011; Mahoney, 2011; Medicines for Malaria Venture, 2021). Examples include Drugs for Neglected Diseases initiative (DNDi), Medicines for Malaria Venture (MMV), the RIGHT Foundation, Gates Foundation, Grand Challenges Canada, Grand Challenges Africa, and Mitacs. Second, while researchers may be committed to the GA principles, the company best suited to take the technology to its next stage in bringing a safe, effective product to market may not be willing to sign onto the GA principles. Technologies developed at universities are usually very early stage, high risk assets, and so finding companies that are willing to accept UBC’s GA conditions on licensing or technology transfer can be a difficult hurdle to overcome. For that reason, UBC has avoided hard and fast rules with respect to GA licensing, attempting to be creative when negotiating with licensing partners to find common ground, one that balances reasonable business requirements with the desire to make technology available to relevant populations. Toward achieving that balance, UBC’s uses the following practices that are well-aligned with the Statement of principles and strategies for the equitable dissemination of medical technologies:

• Make research tools available on a non-exclusive basis. Over the past 2 years, 83% of UBC’s health technology licenses were non-exclusive.
• Refrain from filing patents in least developed nations; unless doing so was more effective at ensuring product delivery to those countries and promoting a sustainable local infrastructure. Over the past 2 years, UBC has sought patents in low and lower-middle income countries on less than 1% of health technologies.^13^ Of the few patents filed in these regions none were on technologies of direct relevance to global health.
• Do not seek royalties on sales to vulnerable populations. This practice was extended to COVID-19 related technology through time-limited, non-exclusive royalty-free licenses, in exchange for the licensee’s commitment to rapidly make and broadly distribute products and services to prevent, diagnose, treat, and contain COVID-19 and protect healthcare workers during the pandemic.
• Implement locally affordable pricing to vulnerable populations. For example, all the medical innovations discussed in Section 3 aimed to follow this approach. In some cases, UBC has the right to grant a non-exclusive royalty-free sublicense to a qualified humanitarian organization.
• Revert to non-exclusive licensing of drugs and medical devices in the relevant jurisdiction if UBC finds evidence that the needs of vulnerable communities are not being met by an exclusive license.^14^
• Gain access to any improvements developed by a licensee of a UBC technology necessary to sublicense to another company that can meet our GA principles.

Finding creative licensing mechanisms to encourage more companies to partner in the development of university technologies under the GA principles is a challenging but vitally important endeavor. Indeed, companies have incentives to enter university partnerships as doing so often aligns with their mandate. For non-profit partners, investigators and institutions often benefit financially in other ways, such as grants and support from philanthropic and donor funding. In the case of for-profit companies, engaging in socially responsible collaborations can help to secure respectable Access to Medicine and ESG (environmental, social and governance) indices, valued by social-minded investors.^15^ In some cases, the technology may have multiple indications, as for Amphotericin B, that allowed the market to be segregated into “high-income” and “low-income” users.^16^ Mechanisms to mitigate arbitrage between the two groups would be required for such a strategy to be effective.^17^ Moreover, the market in the LMIC may be sufficiently large that a for-profit company would be willing to sign onto the GA principles, even with only a small above-cost margin.

## 5 Conclusion

Over the past 15 years, it has become clear that the GA Principles are essential from the perspectives of global health, human rights and social responsibility. A litany of infectious diseases have emerged from Ebola to SARS to Zika to COVID-19 to diseases no longer well controlled, such as highly resistant bacterial infections including methicillin-resistant *Staphylococcus aureus* and carbapenemase producing gram negative organisms. At a profound level, infectious diseases have highlighted our global connectedness. Digitalization of developing healthcare systems and the increasing use of AI are generating a new realm of licensing and sustainability challenges. Improving access to technologies for those who cannot afford modern healthcare improves the health of all (by limiting the spread of disease). Now, more than ever, making healthcare universal and affordable is not only the right thing to do from an ethical and moral point of view, but it is essential to the stability and sustainability of global wellbeing.

As the UBC example illustrates, universities across the globe have an opportunity to make a difference in the race against emerging and re-emerging pathogens through a recommitment to the Global Access Principles. Much has been accomplished but, as Herder et al. (2022) argue, so much more needs to be done in order for university-developed technologies to have a greater social impact on vulnerable populations. While many universities face the same licensing challenges and pressures as UBC, outlined here (Herder et al., 2022; Ramanchdran, 2022), their strategies for overcoming the barriers may be different from UBC’s approach. Efforts are currently underway to bring together research universities in Canada to share their successes and identify best practices for implementing the GA principles. Making university technologies available at affordable prices, in partnerships with the public and private sectors, LMIC governments, local companies and communities, universities can help to improve health outcomes and support a local infrastructure that can sustain these improvements. Indeed, there are multiple hurdles in moving the resources from the lab to the market, but new funding sources directed toward global health, indices for highlighting companies’ charitable and socially beneficial activities, and creative licensing can create incentives for companies to partner with universities in later stages of development and distribution under the GA principles.^18^

## Data Availability

The original contributions presented in the study are included in the article/Supplementary Material, further inquiries can be directed to the corresponding author.

## Footnotes

1 See https://uilo.ubc.ca/about-us/ubc-global-access-principles/history for the history of the GA Initiative at UBC.

2 It should be noted that UBC’s Global Access Principles are not specific to medical technologies. For example, clean-water technologies would be relevant to UBC’s principles and global health, but are not directly medical technologies. However, medical technologies tend to be the innovations that are most readily identified as having global health/access relevance and also the space where UBC’s GA activity has been most prominent.

3 The National Institute of Health (NIH) (https://sharing.nih.gov/other-sharing-policies/research-tools-policy#definition-of-research-tools) defines research tools as “unique research resources that encompass the full range of tools that scientists use in the laboratory, e.g., cell lines, monoclonal antibodies, reagents, animal models, growth factors, combinatorial chemistry and DNA libraries, clones and cloning tools (such as PCR), methods, laboratory equipment and machines … primarily tools for discovery rather than an FDA-approved product or an integral component of such a product.”

4 The National Collaborating Center for Determinants of Health define vulnerable populations as “groups and communities at a higher risk for poor health as a result of barriers they experience to social, economic, political and environmental resources, as well as limitations due to illness or disability.” This definition is consistent with UBC’s practice.

5 Approximately 150–200 inventions are disclosed per year and very few of those have direct relevance to traditional “neglected/infectious” diseases. Further, only a subset is commercially viable and/or find a potential partner that will take the innovation to clinical trials and commercialize.

6 For example, the UILO was involved in setting up the non-profit corporate structure of the Veterans Transition Network (https://vtncanada.org/), an organization that spun-out of UBC research to support veterans at the end of their service. Though not obviously global health, this is an example of support for vulnerable populations.

7 As an institution, UBC applies its GA principles to relevant technology commercialization/mobilization opportunities. Researchers do not formally opt-in (or out) of these principles, but rather self-select projects that are of relevance to global health/access and vulnerable populations.

8
https://autm.net/about-tech-transfer/covid19/covid-19-licensing-guidelines

9 See Mansour, de la Fuente-Núñez, Hancock (2015)

10
https://bmcmedinformdecismak.biomedcentral.com/articles/10.1186/s12911-021-01644-1

11 See Smart Text Analytic Tools (STAT) for analysis of patient-centred communications to strengthen health systems in BC—Health Research BC

12 “Rwanda receives continental award for advancing digital agenda for ending malaria,” *The New Times,* Feb 21, 2023.

13 Under the GA principles UBC may file for a patent in an LMIC, or allow licensees to file in such countries, and use the granted monopoly rights in furtherance of UBC’s GA principles; for example, by ensuring that subsequent “improvement” patents filed by external parties cannot be practiced without a license or sublicense that is compliant with GA principles.

14 Most health-related licenses are non-exclusive because a large proportion are research tools. However, licenses for therapeutics are typically licensed exclusively because of the large amount of investment required to push these assets through clinical trials.

15 See Donald G. McNeil, “Drug Companies are Focusing on the Poor after Decades of Ignoring Them”, *New York Times,* June 24, 2019.

16 Amphotericin B was effective against two diseases: it was an anti-fungal medication as well as a drug for treating leishmaniasis. iCo Therapeutics Inc. received an exclusive license to sell the drug for anti-fungal purposes to high-income populations with the GA commitment to sell at-cost to low-income populations to treat leishmaniasis. Over the past 2 years, 11%–30% of the exclusive licenses on UBC’s health technologies included provisions to promote access to low- and middle-income countries.

17 See Maskus (2001) and Lanjouw (2002) for analyses of parallel importation in pharmaceuticals.

18 See, for example, the discussion in Tannoury and Attieh (2017).
